# Anticancer activity of a sub-fraction of dichloromethane extract of *Strobilanthes crispus *on human breast and prostate cancer cells *in vitro*

**DOI:** 10.1186/1472-6882-10-42

**Published:** 2010-08-05

**Authors:** Nik Soriani Yaacob, Nurraihana Hamzah, Nik Nursyazni Nik Mohamed Kamal, Siti Amalina Zainal Abidin, Choon Sheen Lai, Visweswaran Navaratnam, Mohd Nor Norazmi

**Affiliations:** 1 Department of Chemical Pathology, School of Medical Sciences, Universiti Sains Malaysia Health Campus, 16150 Kubang Kerian, Kelantan, Malaysia; 2 Centre for Drug Research, Universiti Sains Malaysia, 11800 Minden, Pulau Pinang, Malaysia; 3 School of Health Sciences, Universiti Sains Malaysia Health Campus, 16150 Kubang Kerian, Kelantan, Malaysia

## Abstract

**Background:**

The leaves of *Strobilanthes crispus *(*S. crispus*) which is native to the regions of Madagascar to the Malay Archipelago, are used in folk medicine for their antidiabetic, diuretic, anticancer and blood pressure lowering properties. Crude extracts of this plant have been found to be cytotoxic to human cancer cell lines and protective against chemically-induced hepatocarcinogenesis in rats. In this study, the cytotoxicity of various sub-fractions of dichloromethane extract isolated from the leaves of *S. crispus *was determined and the anticancer activity of one of the bioactive sub-fractions, SC/D-F9, was further analysed in breast and prostate cancer cell lines.

**Methods:**

The dichloromethane extract of *S. crispus *was chromatographed on silica gel by flash column chromatography. The ability of the various sub-fractions obtained to induce cell death of MCF-7, MDA-MB-231, PC-3 and DU-145 cell lines was determined using the LDH assay. The dose-response effect and the EC_50 _values of the active sub-fraction, SC/D-F9, were determined. Apoptosis was detected using Annexin V antibody and propidium iodide staining and analysed by fluorescence microscopy and flow cytometry, while caspase 3/7 activity was detected using FLICA caspase inhibitor and analysed by fluorescence microscopy.

**Results:**

Selected sub-fractions of the dichloromethane extract induced death of MCF-7, MDA-MB-231, PC-3 and DU-145 cells. The sub-fraction SC/D-F9, consistently killed breast and prostate cancer cell lines with low EC_50 _values but is non-cytotoxic to the normal breast epithelial cell line, MCF-10A. SC/D-F9 displayed relatively higher cytotoxicity compared to tamoxifen, paclitaxel, docetaxel and doxorubicin. Cell death induced by SC/D-F9 occurred via apoptosis with the involvement of caspase 3 and/or 7.

**Conclusions:**

A dichloromethane sub-fraction of *S. crispus *displayed potent anticancer activities *in vitro *that can be further exploited for the development of a potential therapeutic anticancer agent.

## Background

Cancer is a major public health problem worldwide with millions of new cancer patients diagnosed each year and many deaths resulting from this disease. Chemotherapy remains the principal mode of treatment for various cancers. Tamoxifen, a non-steroidal anti-estrogen drug, is used in the treatment of estrogen receptor (ER)-positive breast cancer patients and as chemoprevention in high risk women [1] but is not effective against ER-negative breast tumours [2]. The anthracycline doxorubicin is frequently used as a chemotherapeutic agent against metastatic breast cancers [3]. Plant alkaloids like docetaxel and paclitaxel are considered highly active chemotherapeutic agents in various cancers including those of the breast and prostate [4,5]. However, development of resistance to chemotherapeutic drugs impedes effective killing of the cancer cells, resulting in tumour recurrence. In addition, patients usually suffer from serious side-effects such as cardiac and other toxicities [6-8].

**Figure 1:**
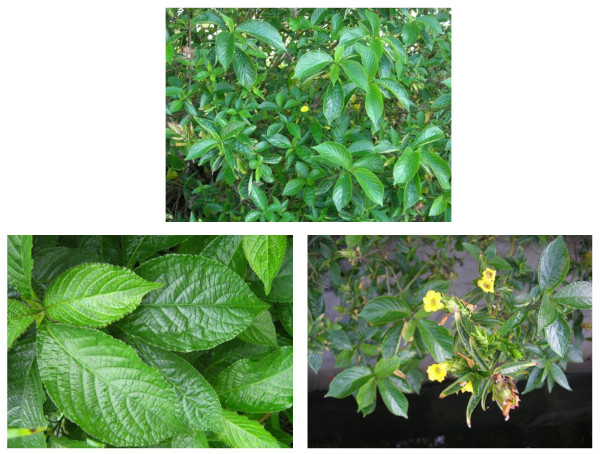
***Strobilanthes crispus***. *Strobilanthes crispus *(Acanthaceae) Blume, also known by its vernacular name of 'pecah beling' or 'pecah kaca', is a flowering shrub distributed throughout the regions of Madagascar to the Malay Archipelago [11]. The plant can either be found wild in scrublands and river banks or cultivated. A mature plant may grow up to a height of 2 m. The stems are grayish in colour, the branches are dark green, the top surface of the leaves are darker green compared to the surface below, oblong to lanceolate in shape with the sides slightly crenated. Both surfaces of the leaves are very scabrid. The corollas are pubescent and yellowish while the calyx segments are covered with patent long and short hairs [12,35].

Natural products have historically and continually been investigated for promising new leads in pharmaceutical development [9,10]. *Strobilanthes crispus *(L.) Blume (*S. crispus*) is distributed throughout the regions of Madagascar to the Malay Archipelago [11] (Figure 1). Traditionally known as 'pecah beling' or 'pecah kaca', the leaves of this plant are boiled with water and used in folk medicine for their antidiabetic, diuretic, anticancer and blood pressure lowering properties [12,13]. However, only a few scientific studies have been conducted to evaluate the reputed efficacy of the plant.

*S. crispus *has high mineral content and contains polyphenols, catechins, alkaloids, caffeine, tannins and vitamins [14] and bioactive components such as stigmasterol and β-sitosterol [15]. The water extract of this plant was reported to contain compounds with very high binding affinity to protein molecules, hence, inhibiting the proliferation of retroviruses [16]. Pharmacological studies have further shown the ability of *S. crispus *in preventing chemically-induced hepatocarcinogenesis in rats [17-19]. Administration of *S. crispus *extract also reduced the severity of hepatic necrosis in rats with diethylnitrosamine- and acetylaminofluorene-induced hepatocellular carcinoma and this was suggested to be due to the inhibition of enzymes involved in metabolic activation of the carcinogens [20]. *In vitro *studies demonstrated that crude methanol (MeOH) extract of *S. crispus *was cytotoxic against HepG2 (liver), Caco-2 (colon) and MDA-MB-231 (breast) cancer cell lines while the chloroform extract was found to be cytotoxic to HepG2 and Caco-2 cells only [15]. These authors also reported that stigmasterol and β-sitosterol isolated from *S. crispus *leaves were cytotoxic to Caco-2, HepG-2, MCF-7 as well as MDA-MB-231 (stigmasterol only) cells. The essential oils of this plant, however, did not display any cytotoxic activity in these cell lines, despite their high antioxidant content [21].

In the current study, the cytotoxicity of various sub-fractions of the dichloromethane (DCM) extract of *S. crispus *was determined and the apoptotic activity of one of the sub-fractions with high cytotoxic potential was further analysed in breast and prostate cancer cell lines. Various sub-fractions of the DCM extract of *S. crispus *were able to selectively induce cell death of breast and prostate cancer cell lines. One of the bioactive sub-fractions, SC/D-F9, was found to be relatively more potent than doxorubicin, paclitaxel, docetaxel and tamoxifen (low dose) *in vitro*, and induced cancer cell death via apoptosis.

## Methods

### Plant material

*S. crispus *(L.) Blume (Acanthaceae) plants were collected from Tasek Gelugur, Pulau Pinang, Malaysia. The plant was authenticated and a voucher specimen of the plant (no. 11046) was deposited at the herbarium of the School of Biological Sciences, Universiti Sains Malaysia.

### Extraction procedures

**Figure 2:**
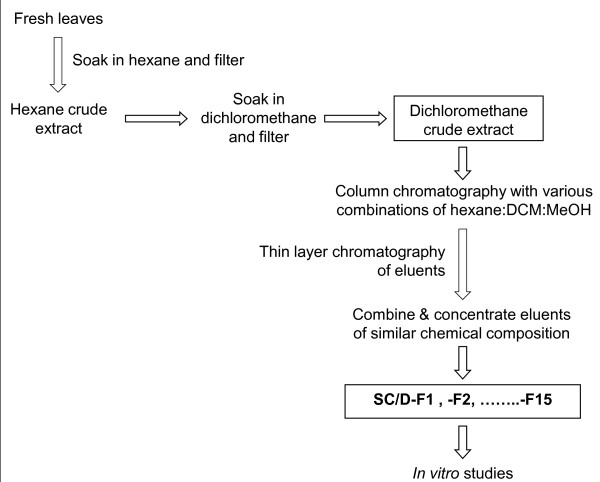
**Flow chart of extraction procedure for *S. crispus***. *S. crispus *leaves were macerated with hexane followed by DCM and fractionated by column chromatography using different combinations of hexane, DCM and MeOH into 15 sub-fractions of differing polarity.

Fresh leaves of *S. crispus *(6 kg) was cut with a mill grinder into fine pieces and macerated with hexane (20 L) for three days at room temperature (*ca*. 25-27°C) (Figure 2). After removing the hexane extract, the residue was then macerated with DCM (20 L) for three days. The extracts were filtered by gravity and the solvents were evaporated under reduced pressure at < 35°C.

### Fractionation of the active plant extract

The DCM extract above was tested and found to be cytotoxic (data not shown) and thus warranted further investigation. The DCM extract (approximately 2 g) was chromatographed on a glass column (50 mm i.d.) packed with silica gel 60, 0.040-0.063 mm (Merck, Darmstadt, Germany) (200 g). Gradient step elution was carried out using a combination of hexane, DCM and MeOH with an initial ratio of hexane-DCM-MeOH, 9:1:0 (v/v/v), followed by 4:1:0, 3:2:0, 2:3:0, 1:4:0, 0:1:0, 0:95:5, 0:9:1, 0:4:1, 0:3:2 and 0:2:3 (v/v/v). The volume of the solvent combination used in each gradient step was 400 ml. Nitrogen gas pressure was applied onto the column at 1 bar to increase the flow of the mobile phase. Eluents were collected in portions of 40 ml. Finally the column was flushed with MeOH.

A small sample of each eluent was evaluated using thin layer chromatography and those eluents which showed similar chemical composition were combined and concentrated under vacuum to yield a total of 15 DCM sub-fractions designated as SC/D-F1, SC/D-F2, SC/D-F3, SC/D-F4, ..., SC/D-F15 (Figure 2).

### Cell culture

A concentrated stock solution of *S. crispus *extracts and fractions was prepared in dimethyl sulphoxide (DMSO) and stored at -20°C until required. Prior to analysis, the samples were diluted in an appropriate growth culture medium with the final concentration of DMSO in culture of ≤ 0.1%.

Human breast (MCF-7 and MDA-MB-231) and prostate (PC-3 and DU-145) cancer cell lines and normal breast epithelial cell line (MCF-10A) were obtained from the American Type Culture Collection (ATCC) (Rockville, USA). MCF-7 and DU-145 cells were cultured in RPMI-1640 medium, MDA-MB-231 cells in Dulbecco's modified Eagle's medium (DMEM), PC-3 cells in Ham F12K medium and MCF-10A in DMEM:Ham F12K (1:1) medium, all supplemented with 100 units/ml penicillin, 100 μg/ml streptomycin (Gibco BRL, USA) and 10% fetal bovine serum (Hyclone, USA) and maintained at 37°C in a humidified atmosphere of 5% CO_2 _in air.

### Cytotoxicity Assay

The cytotoxicity of *S. crispus *DCM sub-fractions was determined using the LDH Cytotoxicity Detection Kit (Roche Diagnostics, Germany) which quantifies the release of lactate dehydrogenase (LDH) from the cells into the culture medium. The cells were seeded in 24-well plates at a density of 1 × 10^5 ^(PC-3, DU-145 and MCF-10A), 2 × 10^5 ^(MCF-7) or 1 × 10^6 ^cells/ml (MDA-MB-231) and grown until about 70% confluence. The medium was then replaced with fresh medium containing 2% fetal bovine serum prior to treatment with the fractions of the extracts for up to 72 hr. The control cells received not more than 0.1% DMSO which was used as a solvent for the extract. Maximum LDH release (high control) was determined by solubilising cells with 1% (w/v) Triton X-100 and spontaneous LDH release (low control) was determined by incubating cells with the medium alone. Cell-free supernatants from the cultures were collected and transferred to 96-well plates for measurement of LDH activity. A reduction reaction of tetrazolium salts, INT, to a red formazan salt was used as an indicator of LDH activity in the supernatant. Absorbance was read at 490 nm and 620 nm reference absorbance by using a microplate reader (Versamax, USA). Results were expressed as % cytotoxicity [(experimental value-low control/high control-low control) × 100]. Effective concentration (EC_50_) values were expressed as μg/ml concentration of the extract that causes 50% cell growth inhibition.

### Annexin V-FLUOS Assay

Apoptosis was determined using the Annexin-V FLUOS Staining Kit (Roche, Germany) in combination with propidium iodide, according to the manufacturer's instructions. Briefly, cells were cultured in chamber slides or T25 flasks at a density of 5 × 10^5 ^cells/ml and allowed to attach overnight, followed by treatment with the SC/D-F9 or anticancer drugs for 24 or 48 hr. Cells were then washed with phosphate-buffered saline (PBS) and incubated with Annexin-V FLUOS labeling solution (containing 2 μl Annexin-V-FLUOS labeling reagent and 2 μl propidium iodide solution in 100 μl incubation buffer) for 10-15 min at room temperature. Analysis was carried out by fluorescence microscopy (Nikon, USA) and flow cytometry (FACS Calibur, Becton-Dickinson, USA). A minimum of 10,000 events were collected for analysis.

### Determination of Caspase 3/7 Activity

Cells were cultured in chamber slides as above for detection of active caspase 3/7 by fluorescence microscopy using the Carboxyfluorescein FLICA Apoptosis Detection Kit (Immunochemistry Technologies, LLC), according to the manufacturer's instructions. Cells were labeled with green fluorescent FAM-VAD-FMK and caspase-3/7 activity was analysed by fluorescence microscopy.

### Determination of Total Phenolic Content

Total phenol estimation was determined according to a previously described method [22]. A 0.2 N Folin-Ciocalteu reagent (500 μl) was added to 1 ml aliquots of the extract (1 mg/ml) and vigorously mixed by vortexing. The mixture was then incubated at room temperature for 3 min. Subsequently, 4 ml sodium carbonate solution (7.5% w/v) was added and the mixture was incubated at room temperature for 60 min. Finally, the absorbance was measured at 760 nm using a spectrophotometer (Shimadzu, Japan) and the measurement was compared to a standard curve of gallic acid. The mean value (± SD) of triplicate analyses is expressed as mg gallic acid equivalents per g plant material (GAE/g).

### Determination of Total Flavonoid Content

Total flavonoid content was determined according to the method of Sakanaka *et al. *[23]. The extract (250 μl, 1 mg/ml) was mixed with distilled water (1.25 ml) and sodium nitrite [75 μl, 5% (w/v)] and incubated at room temperature for 6 min. Subsequently, aluminium chloride solution [150 μl, 10% (w/v)] was added and the mixture was further incubated for 5 min before the addition of sodium hydroxide (0.5 ml, 1 M). Thereafter, distilled water (275 μl) was added and vortexed. Finally, the absorbance was measured at 510 nm using a spectrophotometer and the measurement was compared to a standard curve of catechin. The value (mean of triplicate analyses) is expressed as mg catechin equivalents per g plant material (CE/g).

### Statistical Analysis

Data are presented as mean ± standard deviation (SD) of three independent experiments and statistical significance was determined using Independent Student T test and the SPSS software (SPSS Science Inc.)

## Results and Discussion

**Figure 3:**
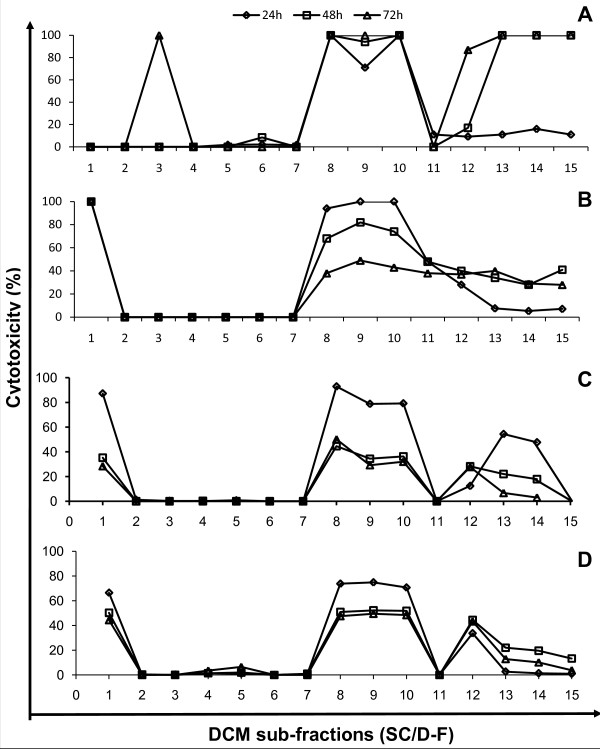
**Cytotoxicity of dichloromethane sub-fractions of *S. crispus *on breast and prostate cancer cell lines**. MCF-7 (A), MDA-MB-231 (B), DU-145 (C) and PC-3 (D) cells were treated with various dichloromethane sub-fractions of *S. crispus *(100 μg/ml) for 24, 48 and 72 hr and cytotoxicity was measured using the LDH release assay. Each value represents the mean of triplicate determinations.

Chromatographic separation of DCM extract of *S. crispus *produced a total of 15 sub-fractions. The cytotoxic potential of these sub-fractions was determined based on the measurement of cytoplasmic LDH enzyme released into the cell culture medium upon damage of the plasma membrane. LDH is a widely used marker in cytotoxicity studies and is a more sensitive indicator of cellular damage compared to the 3-(4,5-dimethylthiazol-2-yl)-2,5-diphenyltetrazolium bromide (MTT) assay [24]. Various sub-fractions of DCM extract of *S. crispus *were found to be highly cytotoxic to the cancer cells while others caused marginal cell death or not at all effective. SC/D-F1 totally killed MDA-MB-231 cells at 48 h but was non-cytotoxic to MCF-7 cells up to 72 hr post-treatment (Figure 3). This sub-fraction was also effective in killing PC-3 and DU-145 cells. SC/D-F3 on the other hand, acted specifically on MCF-7 cells only, causing 100% cell death at 72 hr post-treatment but was not at all effective against the MDA-MB-231 cells. Interestingly, SC/D-F8, SC/D-F9 and SC/D-F10 were found to be consistently highly cytotoxic to all four human breast and prostate cancer cell lines tested, at 24 hr post-treatment. SC/D-F13, SC/D-F14 and SC/D-F15 induced maximum cell death of MCF-7 breast cancer cells within 48 hr but were less potent towards the MDA-MB-231 and the prostate cancer cells.

MCF-7 cell line is estrogen receptor (ER)-dependent and carries the wild type tumour suppressor p53 gene, while the highly aggressive MDA-MB-231 is an ER-independent breast cancer cell line, and is a p53 mutant [25,26]. PC-3 and DU-145 are androgen-insensitive prostate cancer cells [27]. PC-3 is of an aggressive phenotype while DU-145 cells have a more moderate metastatic potential [28,29]. In addition, both prostate cancer cells do not express normal p53 gene. The selective cytotoxic effects of the different DCM sub-fractions observed against the various cancer cell lines tested may be hormone-dependent or -independent, p53-related or influenced by other properties of the cancer cell lines, although these characteristics are yet to be determined.

**Figure 4:**
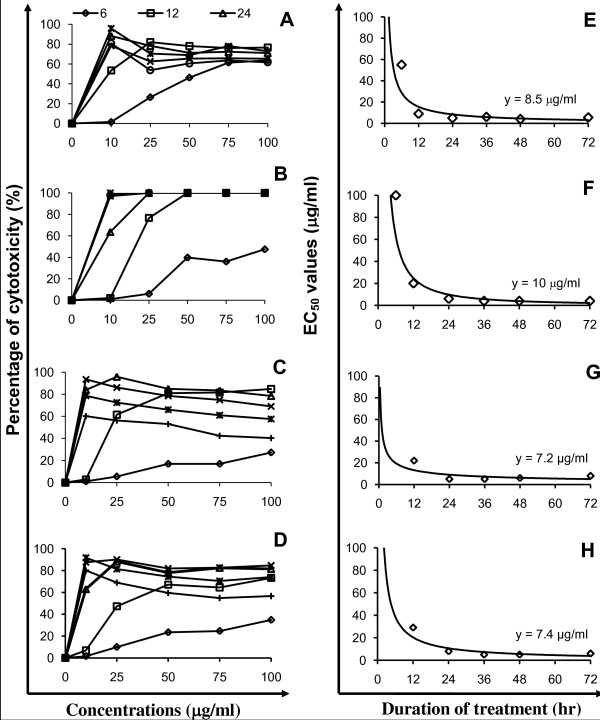
**Dose-response effect and EC_50 _values of SC/D-F9 on breast and prostate cancer cell lines**. Dose- and time-dependent effect of a bioactive sub-fraction of dichloromethane extract of *S. crispus*, SC/D-F9 on MCF-7 (A), MDA-MB-231 (B), DU-145 (C) and PC-3 (D) cells using the LDH assay (prepared in triplicates). The EC_50 _values were plotted against each time point for the determination of the respective constant EC_50 _values (E to H).

One of the most cytotoxic sub-fractions of the DCM extract of *S. crispus*, SC/D-F9, was further analysed to establish its EC_50 _values in the breast and prostate cancer cell lines. Figure 4 shows the dose-response curves and the EC_50 _values for SC/D-F9 in these cells. SC/D-F9 potently inhibited the growth of MCF-7 and MDA-MB-231 breast cancer cells as well as the prostate cancer cells, PC-3 and DU-145, in a time- and dose-related fashion, with low constant EC_50 _values of 8.5, 10.0, 7.4 and 7.2 μg/ml, respectively.

**Figure 5:**
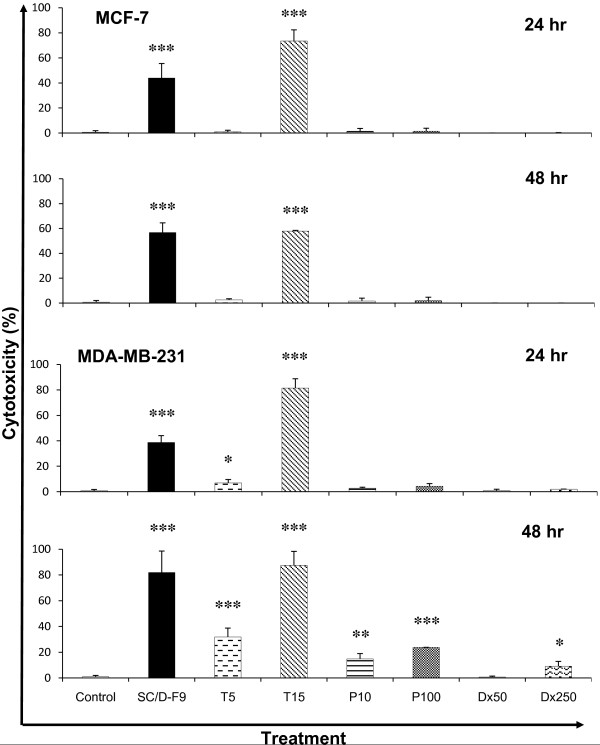
**Comparison of the cytotoxic effect of SC/D-F9 with tamoxifen, paclitaxel and doxorubicin in breast cancer cells**. MCF-7 and MDA-MB-231 cells were treated with 8.5 and 10.0 μg/ml SC/D-F9, respectively, 5 and 15 μM tamoxifen (T5, T15), 10 and 100 nM paclitaxel (P10, P100) and 50 and 250 nM doxorubicin (Dx50, Dx250), for 24 and 48 hr. The control cultures contained the solvent (0.1% DMSO). The percentages of cell death induced were determined using the LDH release assay and each data represents the mean ± SD from three independent experiments. Statistical analysis was determined using the Student T test with * p < 0.05; ** p < 0.01; *** p < 0.001, compared to control.

We further demonstrate that SC/D-F9 is much more effective in inducing cell death compared to some of the commonly used chemotherapeutic agents, tamoxifen, doxorubicin, paclitaxel and docetaxel, in ER-dependent and ER-independent breast cancer cells as well as the androgen-insensitive prostate cancer cells. At the constant EC_50 _value of 8.5 μg/ml, SC/D-F9 caused an average of 44% and 57% MCF-7 cell death in 24 and 48 hr, respectively (Figure 5). Except for the high concentration tamoxifen (15 μM), 5 μM tamoxifen, 10 and 100 nM paclitaxel as well as 50 and 250 nM doxorubicin either did not induce significant death of these cancer cells or displayed much less cytotoxic activity than SC/D-F9, within 48 hr. The MDA-MB-231 cells are more sensitive to SC/D-F9 treatment (10.0 μg/ml) which resulted in 80% cell death within 48 hr, similar to the effect of 15 μM tamoxifen (Figure 5). However, paclitaxel (100 nM) and doxorubicin (250 nM) only killed about 24% and 9% of these ER-independent breast cancer cells. Therefore, we can infer that the cancer cell killing activity of SC/D-F9 is independent of whether the cells express ER or not, indicating that SC/D-F9 has the potential advantage over ER-dependent drugs such as tamoxifen.

**Figure 6:**
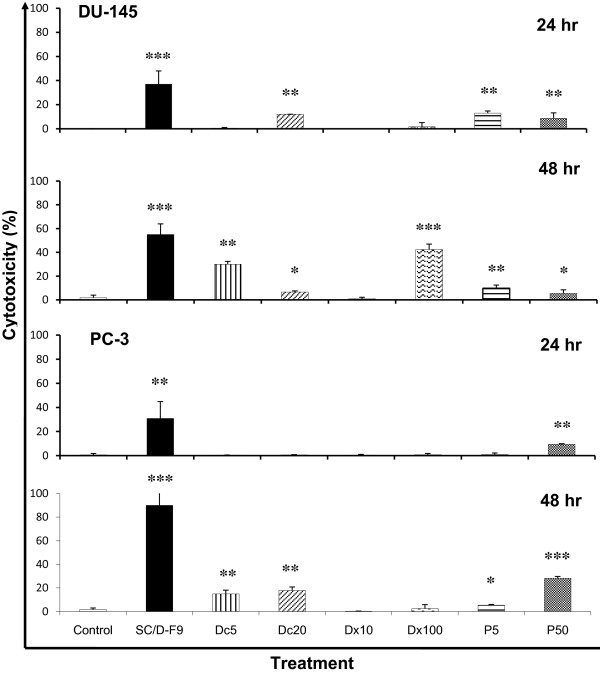
**Comparison of the cytotoxic effect of SC/D-F9 with docetaxel, doxorubicin and paclitaxel on prostate cancer cells**. DU-145 and PC-3 cells were treated with 7.2 and 7.4 μg/ml SC/D-F9, respectively, 5 and 20 nM docetaxel (Dc5, Dc20), 10 and 100 nM doxorubicin (Dx10, Dx100) and 5 and 50 nM paclitaxel (P5, P50) and, for 24 and 48 hr. The control cultures contained 0.1% DMSO. The percentages of cell death induced were determined using the LDH release assay and each data represents the mean ± SD from three independent experiments. Statistical analysis was determined using the Student T test with * p < 0.05; ** p < 0.01; *** p < 0.001, compared to control.

At the constant EC_50 _dose of 7.4 μg/ml, SC/D-F9 effectively killed 90% of PC-3 cells while docetaxel (5 and 20 nM), paclitaxel (5 and 50 nM) and doxorubicin (10 and 100 nM) killed less than 30% of these cells (Figure 6). More than 50% of DU-145 cells were killed by SC/D-F9 (7.2 μg/ml) within 48 hr. In comparison, docetaxel (20 nM) caused about 40% cell death while paclitaxel and doxorubicin resulted in less than 10% cell death. These further indicate that SC/D-F9 is not specifically selective for breast cancer cells only and that its mechanism of action may not be hormone-dependent.

**Figure 7:**
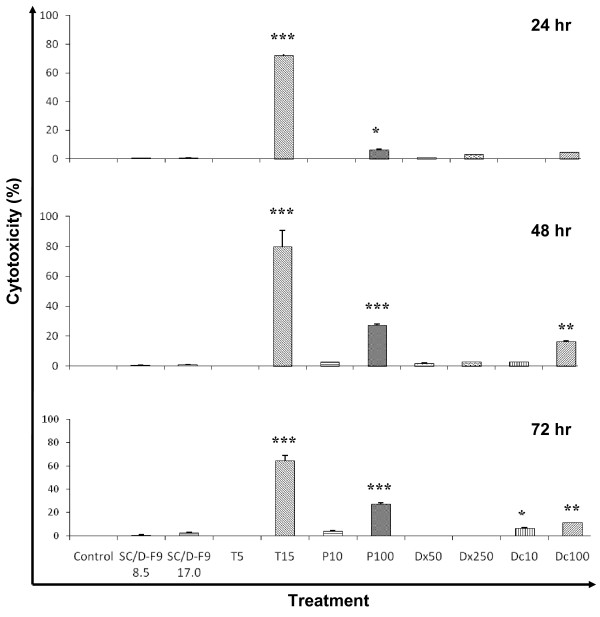
**The effect of SC/D-F9, tamoxifen, paclitaxel, doxorubicin and docetaxel on MCF-10A cells**. MCF-10A cells were treated with SC/D-F9 (8.5 and 17.0 μg/ml), 5 and 15 μM tamoxifen (T5, T15), 10 and 100 nM paclitaxel (P10, P100), 50 and 250 nM doxorubicin (Dx5, Dx250) as well as 10 and 100 nM docetaxel (Dc10, Dc100) for 24, 48 and 72 hr. The control cultures contained 0.1% DMSO. Each value represent the mean ± SD from three independent experiments. Statistical analysis was determined using the Student T test with * p < 0.05; ** p < 0.01; *** p < 0.001, compared to control.

Importantly, we found that SC/D-F9 is non-cytotoxic to the normal breast epithelial cell line, MCF10A, even at twice the constant EC_50 _dose for up to 72 hr while some of the chemotherapeutic agents (especially 15 μM tamoxifen) are found to significantly kill these normal cells (Figure 7). Similarly, crude extracts and essential oils of *S. crispus *leaves were reported to be non-toxic to the normal Chang liver cell line [15,21].

**Figure 8:**
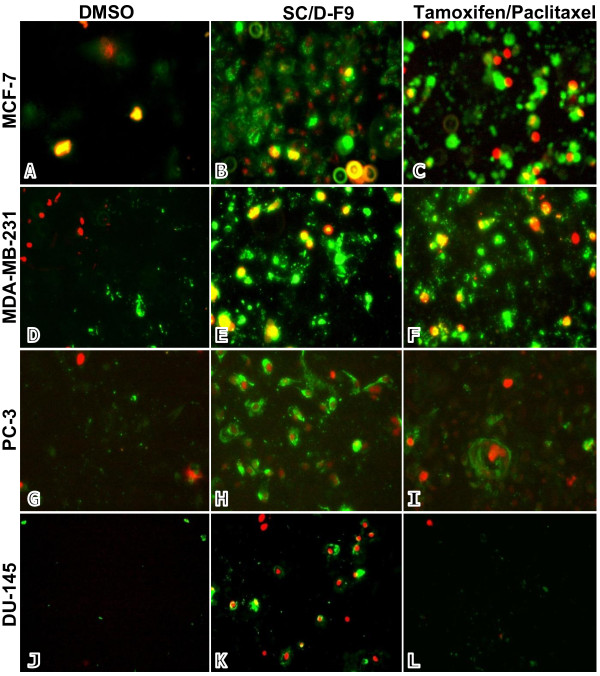
**Apoptosis of breast and prostate cancer cells by SC/D-F9, tamoxifen and paclitaxel**. MCF-7 (A-C) and MDA-MB-231 (D-F) cells were treated with DMSO (0.1%), SC/D-F9 (8.5 or 10.0 μg/ml, repectively), and tamoxifen (15 μM) for 24 hr while the PC-3 (G-I) and DU-145 (J-L) cells were treated with DMSO (0.1%), SC/D-F9 (7.4 and 7.2 μg/ml, respectively) and paclitaxel (50 nM) for 48 hr. The cells were stained with annexin V-FITC antibody (green staining) and propidium iodide (red staining) and observed using a fluorescence microscope (20× magnification).

**Figure 9:**
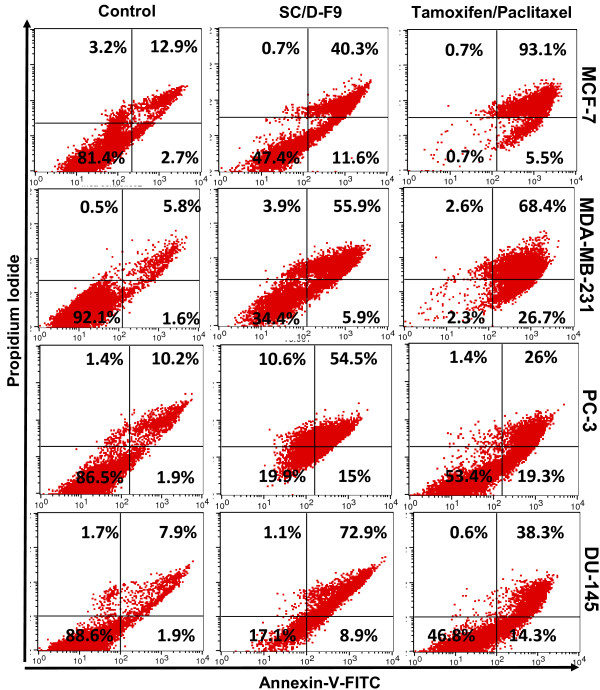
**Percentage distribution of SC/D-F9-induced apoptotic and necrotic breast and prostate cancer cells**. MCF-7 and MDA-MB-231 cells were treated with DMSO (0.1%), SC/D-F9 (8.5 or 10.0 μg/ml, repectively), and tamoxifen (15 μM) for 24 hr while the PC-3 and DU-145 cells were treated with DMSO (0.1%), SC/D-F9 (7.4 and 7.2 μg/ml, respectively) and paclitaxel (50 nM) for 48 hr. The cells were stained with annexin V-FITC antibody and propidium iodide and analysed by flow cytometry.

**Figure 10:**
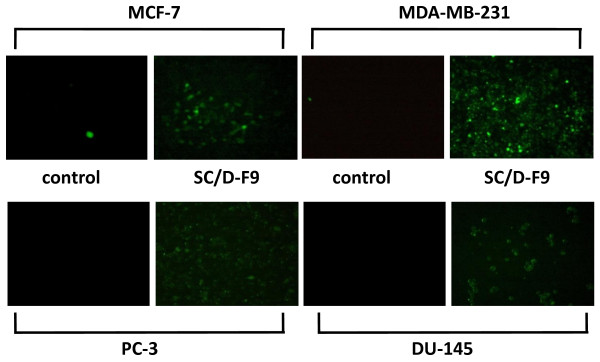
**Activation of caspase 3/7 in breast and prostate cancer cells by SC/D-F9**. MCF-7 and MDA-MB-231 cells were treated with DMSO (0.1%) or SC/D-F9 (8.5 or 10.0 μg/ml, repectively) for 24 hr while the PC-3 and DU-145 cells were treated with DMSO (0.1%) or SC/D-F9 (7.4 and 7.2 μg/ml, respectively) for 48 hr. The cells were reacted with the green fluorescent-labeled caspase inhibitor, FAM-VAD-FMK, and observed under a fluorescent microscope.

Disruption in the balance between the cell-generating process of mitosis and apoptotic cell death can lead to the development of cancer. Blocking cell proliferation and inducing apoptosis are thus considered as important properties of chemopreventive and chemotherapeutic agents [30]. Hence, further analysis was carried out on SC/D-F9 to determine its ability to induce apoptosis. Hallmarks of apoptosis include cell shrinkage, chromatin condensation, nuclear fragmentation and exposure of phosphatidylserine on the surface of cells at the early stages [reviewed in [31]]. Apoptosis in the current study was confirmed by staining cells with the fluorescence-conjugated Annexin-V antibody that binds to phosphatidylserine, and combined with propidium iodide that stains the DNA of cells in their very late stages of apoptosis or of those undergoing necrosis due to compromised plasma membrane permeability. Fluorescence microscopic analysis demonstrates that SC/D-F9 of *S. crispus *induced cell death of all four breast and prostate cancer cells by apoptosis as depicted by strong reaction of these cells with the Annexin V antibody (green), compared to control (DMSO-treated) cells (Figure 8). Significant apoptosis occurs in tamoxifen-treated MCF-7 and MDA-MB-231 cells although much less staining of paclitaxel-treated PC-3 and DU-145 cells is observed. Some propidium iodide staining (red) is also noted in the cells treated with SC/D-F9, tamoxifen and paclitaxel, indicating very late stage apoptosis or necrosis. Figure 9 shows specific percentage distributions of these cells as obtained by flow cytometry. SC/D-F9 effectively caused both ER-positive and ER-negative breast cancer cells to undergo apoptosis within 24 hr. It is also found to induce more apoptosis of the androgen-insensitive prostate cancer cells compared to paclitaxel within 48 hr. To further confirm the apoptotic activity of SC/D-F9, the ability of this sub-fraction to activate the effector caspase 3 or 7 was determined using a potent fluorescent-labeled caspase inhibitor that covalently binds to active caspase within the cells. In all four breast and prostate cancer cells, it can be inferred that apoptosis involves caspase signaling since the caspase 3 and/or 7 was found to be activated by SC/D-F9 although to a lesser extent in the prostate cancer cells compared to breast cancer cells (Figure 10).

Androgens regulate prostate cancer cell growth and differentiation. Current medical therapy for prostate cancer patients includes anti-androgens which inhibit the binding of androgens to the androgen receptor, as well as gonadotrophin-releasing hormone (GnRH) analogues which downregulate GnRH receptors leading to the inhibition of androgen production [32]. This would therefore lead to apoptosis of prostate cancer cells. However, treatment for hormone-resistant prostate cancer is limited and addition of anti-androgens may produce only a transient biochemical response [32]. DU-145 and PC-3 cells are both androgen receptor-positive but androgen non-responsive [27] and hence SC/D-F9 may provide a potential complementary therapeutic agent for hormone resistant prostate cancer.

Analysis of SC/D-F9 showed that it has a total phenolic content of 29.0 ± 0.867 mg/g of gallic acid and a total flavonoid content of 59.0 ± 0.333 mg/g of catechin. These amounts are higher than those reported on some other plant extracts [33,34] and these may contribute to the anticancer effects of SC/D-F9 observed in this study, although this has yet to be confirmed. In addition, it has earlier been reported that *S. crispus *extract has high antioxidant activity that may be attributed to the presence of catechin as well other flavonoids [14]. Since SC/D-F9 is shown to effectively induce apoptosis in androgen non-responsive prostate cancer cells as well as ER-positive and ER-negative breast cancer cells in the current study, the potential of the *S. crispus *plant to be developed further as a cancer therapeutic agent should be explored. Apoptosis is a tightly regulated process controlled by various intracellular signaling molecules involved in different pathways [31]. Work is currently underway to identify the bioactive component(s) in SC/D-F9 to further understand the mechanism of action of *S. crispus*.

## Conclusions

A large number of novel anticancer drugs have been discovered from natural products in the past and new ones are continually being developed. These cytotoxic natural products may be able to play a significant role in treating selected cancers by working in concert with conventional chemotherapeutic drugs thereby improving their efficacy or reducing their toxicity. We have shown that *S. crispus *has potent anticancer activities *in vitro *and could therefore, potentially be a source for a pharmacologically active product suitable for development as a chemotherapeutic agent.

## Competing interests

The authors declare that they have no competing interests.

## Authors' contributions

NSY is responsible for the study design, analysis and data interpretation and manuscript preparation. NH carried out the laboratory work of extraction and fractionation, NNNMK and SNAZA carried out the *in vitro *experiments while CSL oversees the routine laboratory work for the extraction and fractionation of the study. All four participated in the drafting of the manuscript. VN designed the development of the extraction, fractionation and analysis of the bioactive material and edited the relevant part of the paper. NMN participated in the study design, interpretation and edited the final draft of the manuscript. All authors read and approved the final manuscript.

## Pre-publication history

The pre-publication history for this paper can be accessed here:

http://www.biomedcentral.com/1472-6882/10/42/prepub
